# Incurable but treatable: Understanding, uncertainty and impact in chronic blood cancers—A qualitative study from the UK’s Haematological Malignancy Research Network

**DOI:** 10.1371/journal.pone.0263672

**Published:** 2022-02-10

**Authors:** Debra A. Howell, Dorothy McCaughan, Alexandra G. Smith, Russell Patmore, Eve Roman

**Affiliations:** 1 Department of Health Sciences, University of York, York, United Kingdom; 2 Queens Centre for Oncology and Haematology, Castle Hill Hospital, Cottingham, United Kingdom; University of Birmingham, UNITED KINGDOM

## Abstract

**Objective:**

Most blood cancers are incurable and typically follow unpredictable remitting-relapsing pathways associated with varying need for treatment, which may be distressing for patients. Our objective was to conduct a qualitative study to explore understanding among patients with such malignancies, including the explanations given by HCPs and the impact of uncertain trajectories, to generate evidence that could guide improvements in clinical practice.

**Methods:**

The study is set within a population-based patient cohort (the Haematological Malignancy Research Network), in which care is delivered across 14 hospitals according to national guidelines. In-depth interviews were conducted with 35 patients with chronic lymphocytic leukaemia, follicular lymphoma, marginal zone lymphoma or myeloma; and 10 accompanying relatives. Purposive sampling ensured selection of information-rich participants and the data were interrogated using reflective thematic analysis.

**Results:**

Rich data were collected and four themes (11 sub-themes) were identified: 1) Knowledge and understanding of chronic haematological malignancies; 2) Incurable but treatable; 3) Uncertainty about the future; and 4) Treatable (but still incurable): Impact on patients. Patients had rarely heard of blood cancer and many expressed difficulty understanding how an incurable malignancy that could not be removed, was treatable, often for long periods. While some were reassured that their cancer did not pose an immediate survival threat, others were particularly traumatised by the uncertain future it entailed, suffering ongoing emotional distress as a result, which could be more burdensome than any physical symptoms. Nonetheless, most interviewees understood that uncertain pathways were caused by the unpredictability of their disease trajectory, and not information being withheld.

**Conclusions:**

Many participants lacked knowledge about chronic haematological malignancies. HCPs acted to reassure patients about their diagnosis, and while this was appropriate and effective for some, it was less so for others, as the cancer-impact involved struggling to cope with ongoing uncertainty, distress and a shortened life-span.

## 1. Introduction

Arising in blood and lymph forming tissues, haematological malignancies (leukaemias, lymphomas, and myelomas, also known as blood cancers) are collectively the fifth most common cancer grouping in economically developed countries [1, 2]. With diverse aetiologies, treatments, and outcomes, more than 100 subtypes are currently recognized by the World Health Organization (WHO) [3]. Although some of these cancers are potentially curable with intensive chemotherapy (e.g. diffuse large B-cell lymphoma and acute myeloid leukaemia), around 60% are not; the latter typically comprising more chronic or indolent diseases (e.g. chronic lymphocytic leukaemia (CLL), follicular lymphoma (FL), marginal zone lymphoma (MZL) and myeloma) [4]. These malignancies often have a slow manifestation, in which symptoms may be vague, intermittent and commonly seen in benign, self-limiting conditions, particularly in older age groups, meaning cancer is not always immediately suspected [5, 6].

Interestingly, despite being incurable, many indolent blood cancers can be successfully managed, sometimes over many years, on what is considered a remitting-relapsing pathway. This trajectory may include periods of observation (known as ‘active monitoring’ or ‘watch and wait’) usually at diagnosis or when the cancer is in remission, interspersed with treatment at progression or as the disease burden increases (manifested by deteriorating blood results, or new/worsening symptoms), to restore remission. While some patients continue on observation without ever requiring treatment, if/when it is needed (which may occur on multiple occasions) it includes combinations of chemotherapy, radiotherapy, stem cell transplant and targeted agents [7]. Behaviour is known to differ between indolent blood cancers subtypes, with progression almost certain to occur for some (e.g. myeloma), but much less likely for others (e.g. CLL), as is reflected in the five-year relative survival estimates of 48% for myeloma, compared to 86%, 88% and 80% for CLL, FL and MZL, respectively (https://hmrn.org/statistics/survival).

With respect to the experiences of patients with chronic haematological malignancies, much existing literature is limited by the inclusion of individuals with both indolent and acute subtypes, with no differentiation between the two with respect to findings. Such studies have focused on issues such as information satisfaction, decision-making, and quality of life, as well as physician communication styles; identifying considerable scope for improvement [8–14]. Several studies have, however, specifically examined patients with chronic blood cancer subtypes in the last decade or so, with a recent survey identifying poorer diagnostic understanding compared to other malignancies [15]; a worrying issue given the link between information satisfaction and improved quality of life in cancer generally [16]. Other difficulties linked to chronic haematological malignancies include the issue of living with uncertainty, which may be associated with psychosocial problems [17–19].

There is, however, little qualitative evidence about patient knowledge and understanding of chronic haematological malignancies, the explanations given to patients by clinical staff and the impact the uncertain trajectories of these cancers have on those affected over time. To address this, we conducted an in-depth interview study, to generate evidence that could be used to guide improvements in clinical practice. Set within a broader UK National Institute for Health Research (NIHR) programme, this paper is one of a forthcoming series dedicated to examining patient experiences of chronic blood cancers, including information needs and preferences for involvement in decision-making.

## 2. Methods

Methods are described in accordance with COREQ [20].

### 2.1 Setting

This qualitative study was set within the infrastructure of the UK’s Haematological Malignancy Research Network (HMRN: www.hmrn.org), a population based patient cohort initiated in 2004 to inform research and clinical practice; locally, nationally and internationally [7]. HMRN’s configuration, methods and approvals have been published [21]; and the present study has additional ethical support (REC:16/LO/0740). Briefly, HMRN has a catchment population of ~4 million, with a similar socio-demographic profile to the UK as a whole; and patient care is provided by a unified clinical network (14 hospitals), working to national guidelines. All haematological malignancies in the study area are diagnosed by a single laboratory (the Haematological Malignancy Diagnostic Service: www.hmds.info), using the latest ICD-O classification [3]. Patients enter the cohort at diagnosis (~2,400 annually), and have diagnostic, prognostic and clinical data (including all treatment and responses) collected from their medical records.

### 2.2 Sampling strategy

In-depth interviews were conducted with patients from HMRN’s established Partnership (https://yhhn.org/partnership), who had agreed they could be contacted for research purposes. Purposeful sampling was utilised, in which patients were intentionally selected based on their demographic and diagnostic characteristics, in the likelihood of them being ‘information-rich’ sources, able to provide data that were relevant to the research aims [22]. In this context, initial inclusion criteria included: diagnosis of CLL, FL, MZL or myeloma (reflecting the spectrum of chronic cancers) in men and women close to the median diagnostic age for each subtype. Variation was then introduced by socio-economic area, age strata and time since diagnosis, to capture more diverse experiences [22, 23]. The number of interviews conducted was guided by the concept of information power [24, 25], which aligns with our analytical method, outlined in 2.4.

### 2.3 Recruitment and data collection

After checking with NHS staff that patients were alive, and well enough to participate, potential interviewees were sent an information sheet and asked to contact the research team if they wanted to take part, and to ask a relative/friend to join the interview, if they wished. Interviews were conducted February to October 2019, at a time and place chosen by the patient, usually their home. Informed written consent was obtained (permitting use of direct quotes) following assurances about confidentiality and anonymity, and the opportunity to ask questions. Interviews were conducted by an experienced researcher, lasted ~90 minutes and were digitally audio-recorded. Patients were asked to tell their own story from diagnosis, with a topic guide used to direct questioning (**S1 File**). Recordings were transcribed externally, then checked, corrected and anonymised by the interviewer.

### 2.4 Data analysis

Data analysis was conducted by the interviewer and a second researcher utilising reflexive thematic analysis, a method commonly used in studies seeking to identify patterns of meaning (‘themes’), which does not adhere to any particular theoretical stance [26, 27]. The initial step in this approach involved familiarization and engagement with the data by active (i.e. analytical, critical) reading and re-reading of the transcripts, while constantly attempting to interpret the information provided. This was followed by the generation of useful, meaningful codes, by means of a fluid, organic, active process in which codes evolved, were renamed, divided, collapsed and/or deleted [25]. The next step was to search for and develop themes from the codes, which were then reviewed within a thematic map, before finally being defined and named.

## 3. Results

**Fig 1:**
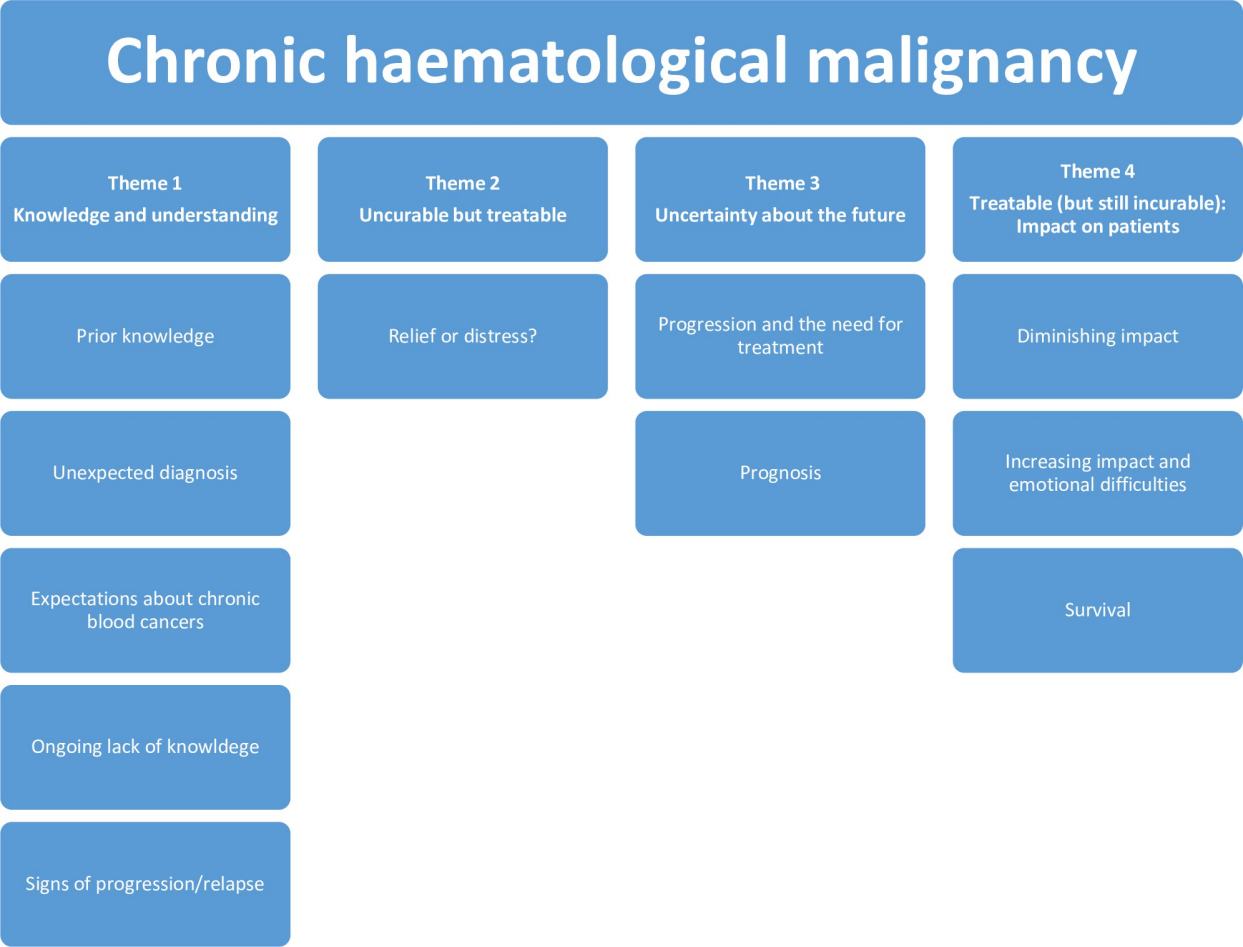
Hierarchical map of themes and sub-themes.

Thirty-five patients were interviewed, 10 with a relative present (contributing to varying degrees). Pathway overviews and participant characteristics, based on routine HMRN data collection from medical records are shown in **Table 1**. The majority were aged in their sixth or seventh decade at interview, nineteen were male, and most resided with a relative, with three living alone. Twelve had myeloma, ten CLL, eight FL, and five MZL. Rich data were accumulated and reflexive thematic analysis resulted in the identification of four themes and 11 sub-themes. Key themes included: 1) Knowledge and understanding of chronic blood cancers; 2) Incurable but treatable; 3) Uncertainty about the future; and 4) Treatable (but still incurable): Impact for patients. Each theme is described below with sub-themes. Quotations are shown in italic and linked to participant numbers (e.g. P1 for the patient, P1R for P1’s relative) and diagnosis. **Fig 1** depicts the hierarchy of themes and sub-themes.

**Table 1:** Characteristics of interviewees.

ID	Diagnosis^1^	Year of diagnosis	Sex	Age at Diagnosis (Years)	Age at interview (Years)	Lived with relative or alone	Relative present at interview	Treatment line(s) preceding interview^2^^,^^3^					
								1^st^	2^nd^	3^rd^	4^th^	5^th^	6^th^
P1	CLL	2015	F	64	67	Relative	-	Observation	-	-	-	-	-
P2	MZL	2004	M	55	69	Relative	-	Observation	Chemotx	Observation	-	-	-
P3	CLL	1997^4^	M	40	62	Relative	-	Observation	Chemotx	Observation	-	-	-
P4	MZL	2016	F	57	60	Alone	-	Observation	Chemotx	-	-	-	-
P5	MZL	2017	F	54	56	Alone	-	HPE	Observation	-	-	-	-
P6	CLL	2011	F	68	75	Relative	Yes	Observation	Chemotx	Observation	-	-	-
P7	CLL	2013	M	63	68	Relative	Yes	Observation	Chemotx	Observation	-	-	-
P8	FL	2016	F	70	72	Alone	-	Chemotx	Radiotx	Observation	-	-	-
P9	CLL	2014	M	80	86	Relative	-	Observation	Chemotx	-	-	-	-
P10	FL	2011	M	66	73	Relative	-	Observation	Chemotx	Chemotx	Chemotx	-	-
P11	Myeloma	2014	M	56	65	Relative	-	Observation	Chemotx	Observation	-	-	-
P12	MZL	2014	M	69	73	Relative	-	Observation	Chemotx	-	-	-	-
P13	CLL	2018	F	56	57	Relative	-	Observation	-	-	-	-	-
P14	Myeloma	2015	M	56	60	Relative	-	Steroids	Radiotx	Chemotx	Chemotx	Chemotx	SCT
P15	FL	2016	F	72	75	Relative	-	Observation	Chemotx	-	-	-	-
P16	Myeloma	2017	M	64	66	Relative	-	Chemotx	Chemotx	Chemotx	SCT	Observation	-
P17	FL	2016	F	64	67	Relative	Yes	Observation	-	-	-	-	-
P18	Myeloma	2016	M	60	63	Relative	-	Chemotx	Chemotx	Chemotx	SCT	Observation	
P19	FL	2016	F	51	54	Relative	-	Steroids	Chemotx	Chemotx	Observation	-	-
P20	CLL	2015	M	71	74	Relative	Yes	Observation	-	-	-	-	-
P21	Myeloma	2016	M	67	70	Relative	Yes	Steroids	Chemotx	Chemotx	Chemotx	SCT	-
P22	CLL	2016	M	69	72	Relative	Yes	Observation	Clinical trial	Observation	-	-	-
P23	Myeloma	2016	F	60	63	Relative	-	Observation	-	-	-	-	-
P24	FL	2015	M	53	57	Relative	-	Steroids	Chemotx	Radiotx	Observation	-	-
P25	FL	2015	F	63	67	Relative	-	Chemotx	Chemotx	-	-	-	-
P26	Myeloma	2015	F	68	72	Relative	-	Observation	-	-	-	-	-
P27	CLL	2015	M	71	75	Relative	Yes	Chemotx	Observation	-	-	-	-
P28	Myeloma	2015	M	59	63	Relative	-	Steroids	Chemotx	Chemotx	SCT	Clinical trial	Chemotx
P29	CLL	2016	F	70	73	Relative	-	Clinical trial	Observation	-	-	-	-
P30	Myeloma	2017	M	70	72	Relative	Yes	Observation	-	-	-	-	-
P31	Myeloma	2017	M	71	73	Relative	Yes	Radiotx	Steroids	Chemotx	Observation	-	-
P32	MZL	2017	F	60	62	Relative	Yes	Observation	Chemotx	Observation	-	-	-
P33	Myeloma	2016	F	53	55	Relative	-	Chemotx	Chemotx	SCH	Observation	-	-
P34	FL	2015	M	53	57	Relative	-	Steroids	Chemotx	Chemotx	Chemotx	-	-
P35	Myeloma	2017	F	55	57	Relative	-	Chemotx	Chemotx	Chemotx	Chemotx	SCT	Observation

^1^ CLL: Chronic lymphocytic leukaemia; FL: Follicular lymphoma; MZL: Marginal zone lymphoma.

^2^ Chemotx = Chemotherapy; HPE = Helicobacter pylori eradication; Radiotx = Radiotherapy; SCT = Stem cell transplant (all autografts); SCH = Stem cell harvest (shown for P33 because this patient’s SCT was cancelled as it was considered risk by clinical staff and the patient).

^3^ Does not include supportive care (e.g. blood product transfusions, plasma exchange, bisphosphonates, cell mobilization products).

^4^ Patient was diagnosed pre-HMRN; pathway data collected at interview.

### Theme 1, knowledge and understanding of chronic blood cancers

This theme contains five sub-themes that focus on patient knowledge, understanding and expectation regarding blood cancers.

#### Sub-theme 1, prior knowledge

Many people said they had no prior knowledge of their malignancy type, with one saying he knew about ‘*standard cancers*’ but not myeloma (P16). In the context of CLL, P13 said she had known ‘*nothing*’ about her disease; and P29 said she had known leukaemia was a ‘blood cancer’, but no more. P30 said he *‘didn’t even know the word myeloma until I went to the doctors’*. Patients did not always immediately understand the nature of their chronic blood cancer, but initially reported focusing on specific phrases such as ‘not curable’ and ‘leukaemia’, which could be distressing, incurring shock and fear, particularly as there was little awareness about indolent and acute subtypes, or the different pathways and outcomes associated with each of these.

#### Sub-theme 2, unexpected diagnosis

Some patients had been diagnosed incidentally and otherwise considered themselves well, so were confused to discover they had cancer. In patients with myeloma, for example, P21 was diagnosed at a routine check-up *‘by accident’;* and P16 said his diagnosis came ‘*out of the blue*’ as he considered himself fit and active. Others had only minor symptoms and didn’t always feel ill, with P22 (CLL) explaining his surprise at being told he was ‘*a very poorly man’* at his first appointment, as his only prior symptom was tiredness; P15 (FL) declaring ‘*I wasn’t ill…it was just the lumps I found’*; and P27 (CLL) saying apart from difficulty walking, he felt fine.

This was augmented in a number of patients, as they had actually attended medical appointments for what they presumed to be unrelated symptoms, but which were later attributed to their blood cancer. Examples include diagnosis following screening to assess breast lumps (P15: FL, P20: CLL); a GP visit for leg pains (P29: CLL); an ENT appointment for a neck lump (P17: FL); and a scan for osteoporosis (P35, myeloma). Conversely, however, some individuals had heard of their cancer and knew of its clinical signs, with one saying that the *‘jigsaw fell into place’* (P12: MZL), as he had thought his symptoms were due to lymphoma.

#### Sub-theme 3, expectations about chronic blood cancers

A number of patients struggled to understand the characteristics of their cancer and why it could not simply be removed. In this context, P5 (MZL) described her main barrier being that what seemed like a stomach problem couldn’t be treated with a simple *‘zap’*; she did, however, accept that her cancer was different to the type you could just *‘get rid of’*. Similarly, P13 (CLL) said she had found it hard to understand she had leukaemia because the first symptom was a lump in the armpit, which led her to presume that: *‘if it’s a lump they can just take it out’* and resulted in ‘*a bit of a shock*’ when she found this was not possible as the cancer was in her blood, so in her *‘whole body’*.

Based on their knowledge of other cancers, most people expected to start treatment immediately after diagnosis, whereas many were initially observed. While this satisfied some, particularly if a HCP had clearly explained the underpinning rationale, many found it difficult to comprehend. P26 (myeloma), for example, said: *‘it’s hard to understand you have something that is so…really frightening…yet nothing happens’*. P15 (FL), said she found it challenging to understand and accept and was *‘not prepared’* for the situation; she was also unclear about the circumstances that would lead to her starting treatment, saying: ‘*that was always a question I had; how doctors would know when to start treatment*: *it was almost like you had to become ill…*.’. Similarly, P6 (CLL) and her husband described difficulty comprehending that *‘nothing was being done’*, whilst they just waited for things to get worse before: *‘they’ll sock it (treatment) to you’*.

#### Sub-theme 4, ongoing lack of knowledge

While some later came to have a good grasp of their illness, others didn’t seem to reach this point and were still unclear about their malignancy a number of years post-diagnosis. P32 (MZL), for example, said she could not discuss her diagnosis with family, partly because she didn’t really understand it: *‘we’ve never ever… we’ve got a son and a daughter*, *we’ve never told them because really*, *we don’t know what we’re talking about…’*. Knowledge and understanding was found to impact on diagnostic disclosure, with P20 (CLL) saying he told his immediate family, but not his friends as he believed they wouldn’t comprehend why treatment had not started: *‘I don’t publicise it’*. This was echoed by P25 (FL), who said others didn’t understand the characteristics and impact of her cancer.

#### Sub-theme 5, signs of progression/relapse

Several patients demonstrated clear knowledge of the signs of progression/relapse and how to respond: *‘(I) keep a look out and get in contact with them (HCPs) when…I get any…B symptoms of night sweats…itchy skin*, *tiredness*, *you know*, *problem with breathing and all that’* (P34: FL); with P25 (FL) saying she would ‘*know straightaway if something was wrong*’. Others were, however, worried about their lack of awareness. P19 (FL) for example, said that initially she hadn’t been told what to look for, and was ‘*not convinced that I would know if it was back*’. Having responsibility for recognising potential signs of progression and deciding when to report these was difficult for some, with P35 (myeloma) explaining: *‘That’s why it’s so complicated*. *So*, *it’s kind of down to me to tell them if I don’t feel right and I just find*, *that’s just a massive pressure’*.

### Theme 2, incurable but treatable

This theme focuses on the information provided by HCP to promote understanding among patients and families about the characteristics of the chronic haematological malignancies; it also contains a sub-theme about response to this. Patients often mentioned that HCPs had referred to their cancer as being incurable but treatable. Phrases HCPs were said to use include: ‘*it’s very treatable’* (21R, myeloma); and ‘*there’s no cure but it is not life-threatening*’ (P20R, CLL). Similarly, P34 (FL) described being told he had cancer, but that it was: ‘*…low grade…slow growing but harder to get rid of*…*an incurable cancer; (that) the chemo was probably going to be effective in some way*, *but… I’d never be in full remission’*.

A number of people said their HCP likened their diagnosis to living with a chronic illness, with comparisons made to ‘*diabetes*’ (P15: FL), and ‘*similar to me COPD’* [sic] (P25: FL). In terms of prognosis, several others said that reassurance emerged via HCPs implying they would die from other causes, not the blood cancer itself, recounting phrases such as: *‘(you) could actually die with it*, *not because of it’* (P7, CLL). P32 (MZL), described being told she could expect a lifespan that was *‘same as anybody else’*. P32R went on to say *‘…it’s the stigma*, *with the word cancer and that*. *Everybody thinks it’s a death sentence*, *don’t they*? *So (doctor) sort of said it*, *there’s something wrong with you*, *but you can live with it*. *It wasn’t life threatening or anything like that…’*.

**Sub-theme 1, relief or distress?** Many interviewees described feeling relief at hearing their cancer was incurable but treatable, with P7 (CLL) considering this: ‘*a big ray of hope in the distance’*; and P3 (CLL) feeling *‘positive’* after his haematologist told him he would certainly be attending clinic ten years hence. P12 (MZL) said that after being told *‘it’s more likely you’ll die with it than of it…*.*that phrase settled me…’*. Another was similarly reassured when their consultant said: ‘*…this could take weeks to develop*, *months*, *decades…go and live your life’* (P26: myeloma). P17 (FL) described being ‘*in a bit of state of shock at diagnosis*’, but relieved when the haematologist said: *‘we can treat this’*. The relative of P22 (CLL), said she and her husband coped by focusing on such positive phrases.

While some patients were reassured, however, others were deeply troubled. P35 (myeloma) for example, said progression was always on her mind: ‘*it’s just horrible being in this position where you know (the paraprotein) it’s creeping up’*. Similarly, P34 (FL), said: *‘it’s hard not to think that everything is related to lymphoma*. *Any time something happens to me I’ve got it in the back of my mind*, *what’s this ache I’ve got*?*’*. P17 (FL) described the sudden appearance of a neck lump (later diagnosed as a cyst), saying: *‘you obviously think it’s something to do with the lymphoma’*. The need for counselling, or psychological/emotional support was noted by some patients, and also family members. P4 (MZL), P13 (CLL) and P19 (FL), for example, reported accessing such services to help them manage diagnostic distress; although P12 (SMZ), said his diagnosis didn’t affect him ‘*psychologically*’ as he had a ‘*low-grade type*’. This issue of distress is picked up in greater detail in Theme 4, Sub-theme 2.

### Theme 3, uncertainty about the future

Uncertainty generally pertained to the occurrence and timing of cancer progression, the need for treatment, and prognosis, as described within the two sub-themes below.

#### Sub-theme 1, progression

Although patients were told progression might never occur, they were aware it could still happen at any time and on numerous occasions, with the need for multiple lines of increasingly intensive chemotherapy. In the context of myeloma, P28 said ‘*they’ve wanted to put me on a 4*^*th*^ *line of treatment…*.*my light chains are very high…’*. Changes were said to happen slowly or rapidly, and could lead to altered treatment plans. P18 (myeloma), for example, described gradually *‘getting more and more breathless’* in the post-transplant period as he relapsed, with further chemotherapy given prior to a planned second transplant, which was then abruptly *‘ruled out’* as his *‘free light chains had rocketed back up again’*.

Uncertainty about the timing of progression/relapse was said to be clearly communicated at various time-points on the pathway. At diagnosis, for example, P20 said his CNS *‘went through it very thoroughly’* explaining that his CLL may progress, but that *‘nothing might happen during (his) life-time…you might live with it as long as you live’*; and P30 (myeloma) noted how one doctor *‘went straight to the point’*, telling him *‘sometime or other chemo will have to come in*, *but he didn’t say when’*. Uncertainty about future progression/relapse was also conveyed post-treatment, as noted by P34 (FL) whose HCP *‘basically explained…how you cannot predict what’s going to happen*. *It may never*, *ever come back*. *It may come back tomorrow*. *It’s just completely uncertain and that’s what you have to have in your head…’*. Similarly, P10 (FL), described how after second-line chemotherapy, his consultant had said the cancer: *‘had gone completely*, *but could come back 5 or 10 years down the line (as there wasn’t) 100% guarantee that it won’t*, *but in all honesty*, *we think it probably will at some stage come back…it’s a raffle really…you could be lucky or it could come back’*. P16 (myeloma) said *‘(HCPs) advise you…be prepared it could return…I know mine will…’*.

#### Sub-theme 2, prognosis

With respect to prognosis, patients described being told that their survival duration was also unclear. Importantly, however, there was often recognition and understanding that this reflected genuine clinical uncertainty, due to the unpredictability of their cancer, rather than the withholding of information by HPCs **(Box 1)**. P11 (myeloma), for example said he had reached ‘*a plateau*’ and that his clinicians had said: ‘*some people stay on that plateau for quite a long time*, *others don’t*’. This patient seemed to understand that doctors couldn’t be certain about individual patients, and that nobody can ‘*really know what’s in store down the line*’. P25 (FL) described how she wanted to know more about her prognosis including: *‘what is the likelihood of (the cancer) coming back…what’s the odds*?*’* and ‘*how many people live to a ripe old age and die of something else*?*’* but compared this to asking *‘how long is a piece of string*?*’*; and P3 (CLL) explained that having an uncertain pathway meant it was difficult to know when was the right time to ask about prognosis.

**Box 1. Reflections on genuine clinical uncertainty**

○ *‘(HCPs) couldn’t say for sure what my prognosis was because they really didn’t know… everyone is different*, *that’s what I learned’* (P3: CLL)

○ *‘(HCPs) just don’t know how it will evolve in your body’* (P4: MZL)

○ *‘there aren’t any answers…you’ve just got to wait and see’* (P7R: CLL)

○ *‘everyone is different and so it’s difficult to say*, *well this is going to happen…it’s not that certain’* (P15: FL)

○ *‘(HCPs) can’t give you a timescale…you accept the worst and hope for the best’* (P16: myeloma)

○ *‘there are a lot questions that people just don’t know the answers to’* (P18: myeloma)

○ *‘(HCP) just said “we don’t know…”*, *they’ve never actually said “the average is 6 years or the average is… [before relapse]” I don’t think they can’* (P19: FL)

○ *‘(HCP said) we can’t give you an answer (about prognosis)*, *we don’t know…everybody is different*, *which I can accept that’* (P25: FL)

○ *‘myeloma is a very individual disease…you get the same treatment*, *same this*, *same that*, *but you have different outcomes’* (P28: myeloma)

### Theme 4, treatable (but still incurable): Impact on patients

Having a treatable but incurable chronic haematological malignancy, affected patients differently, with the diagnosis gaining or losing impact over time, and some individuals experiencing particular emotional difficulties coping with uncertain future pathways, as depicted in the sub-themes below.

#### Sub-theme 1, diminishing impact

For some, the impact of their indolent blood cancer diminished as they became more accustomed to it, especially if they had not required any treatment, and were being monitored less often. This was particularly apparent in CLL, but also to a lesser extent other diagnoses, with one patient (P26, myeloma) starting with 3 monthly monitoring, which reduced to checks every 6 months, before being replaced by telephone appointments. In another example, P29 said she had spoken to her consultant about the future and been told ‘*right at the beginning*, *maybe 5 years*, *maybe more*’; 3 years post-diagnosis at interview, she said: ‘*so I’ll just keep going’*, saying she didn’t want to dwell on her disease, as keeping positive helped her cope.

#### Sub-theme 2, increasing impact and emotional difficulties

For many, having a chronic haematological malignancy had a significant impact on their life. One such group included patients for whom severe emotional distress appeared to be ongoing, causing greater difficulty than any physical effects from the cancer. Such anxiety was clearly portrayed by P35 (myeloma), who said: ‘*…you’re at different stages all the time*, *and I’m in a bit of a difficult [stage] I think it’s like fear of not knowing what’s going on is harder than like*, *if someone says*, *you’ve got to have this and [you can] psych up for it*. *I’m just in an awful time for me*, *but then again*, *I’m constantly wrangling with myself*, *because then I just think*, *I’m just so grateful to be here and I do have treatment options*, *you know*, *it could be worse…and my quality of life is good at this point and I don’t want to waste it by [being] up and down*. *Oh*, *I’m so anxious*, *you know*, *I really don’t want to do that*. *I just want to get on*. *It’s bloody hard though’*. Similarly, P25 (FL) described distress caused by wondered how long her cancer would be *‘manageable’*, comparing her situation to *‘Russian roulette (where) someone has got a gun against my head…’*. The same patient, who had attended the haematology clinic regularly since diagnosis, reported being anxious when she didn’t receive her usual appointment and *‘kept ringing up’*, only to be told she was on a waiting list. She said she just needed reassuring that she was *‘alright’*, but felt her HCPs were simply ‘*waiting for me to die’*.

#### Sub-theme 3, survival

Despite being treatable, chronic blood cancers are generally considered incurable, and can potentially effect survival, which was of great concern to some, including younger patients, such as P28 (age 59 at diagnosis; myeloma): *‘it’s a non-curable cancer… certainly*, *it’s treatable*, *but nonetheless*, *that was kind of a big shock in itself*, *a huge shock (finding) 50% of people survive 5 years’*. In an ‘*unforgettable’* exchange with an HCP, P4 (age 57, MZL) recounted being reassured that her cancer was treatable, but then learning it could significantly limit her life expectancy: *‘the first thing (HCP) said to me was “you might only live 5 years with it…”*. *I just couldn’t take it in…you’ve just been told you’ve got cancer and she’s saying “you might only live for 5 years*!*”‘*. *S*ome older patients viewed their prognosis in the context of their life-span, however; P15 (FL, age 72), for example, saying: ‘*10 years*, *which at my age is more than you could hope for’*.

Interestingly, P35 (myeloma) noted undue optimism from clinical staff about her treatment and prognosis: *‘They kind of just acted in a positive way*. *They don’t say*: *“it might not work”*. *They’re just being really positive*, *but…I was in the unfortunate situation of knowing someone really well who had (myeloma)*, *and he’d had a really bad time and none of the treatment worked’*. P35 went on to describe having asked: ‘…*can I live to be an old person*? *And (nurse) said…I’ll never forget it*, *it’s in my mind a lot*, *she said*, *“you might have to re-evaluate what you mean by old”*, *and it really sticks in my mind but I couldn’t bring myself to ask any more questions [became upset]’*. She then demonstrated a mix of fear and hope, saying: ‘*I’ve never dared ask how long I might live and things like that*, *because they don’t know*, *because like*, *what works for one person doesn’t work for another*, *and you get these people who get long remissions*. *I always have a few questions*, *but I don’t ask the things that are sort of on my mind*. *It’s just too big’*.

Maintaining optimism was considered important by P35 (above) and other interviewees. P24 (FL) said it was important for doctors to give patients hope, and for patients to maintain a positive mental outlook. Patients themselves often placed their hope in new therapies, with P34 (FL) saying he hoped his prognosis had improved since diagnosis: *‘treatments have changed… and obviously it’s going to be a lot better outcome (now)*. *I know that the outcome would have been different if I hadn’t have had (drug)*… *you know*, *I don’t think I would have been told 5 years*. *But now*, *who knows*. *They told me if I went now*, *they’d give me a different… prognosis*. *They’d be looking at what I am now…going forward…’*. P11 (myeloma) said: *‘they are constantly improving medication’*; and the relative of P27 (CLL) reported their consultant saying *‘things are moving on all the time… we can re-treat’*, which gave them *‘a lot of confidence’*.

## 4. Discussion

This study contributes novel evidence about the experiences of patients with chronic haematological malignancies. Specifically, we identified a distinct lack of knowledge about such cancers among patients, HCP communication strategies that often aimed to reassure individuals about their indolent diagnosis, and an immense amount of uncertainty about the future. The resulting impact on patients varied, with some feeling relieved that although their chronic cancer may not be curable, it could be treated (if treatment was ever required), while others struggled to grasp and deal with this. Living with uncertainty often caused marked ongoing emotional distress, even among patients with the most indolent diseases, who were asymptomatic and did not require treatment; and in many cases this appeared more burdensome than any physical consequences of the cancer.

The unusual pathways of chronic haematological malignancies clearly impacted on the well-being of some patients. Compared to other cancers, for example, where relapse may only need to be considered once or twice, individuals with chronic blood cancers may have to face this repeatedly, on a third, fourth or subsequent occasion, across their remaining life. This is also a crucial difference between the chronic subtypes targeted in the current study and the more aggressive entities that may be potentially curable with intensive treatment; after which (similar to many other cancers) a distinct ‘survivorship’ phase begins. This marks another divergence, as traditional concepts of survivorship denote a phase ‘beyond’ treatment [28–30], a time-point never reached on the remitting-relapsing pathway of chronic blood cancers, meaning resources and national initiatives set-up to meet long-term needs are not always applicable to these patients.

Although communicating information about uncertain pathways is a major component of existing good clinical practice, which was clearly appreciated by participants, some continued to struggle with this constantly being a part of their lives. Described as an ‘ever-shifting perspective between illness and wellness’ in the context of myeloma [18], this situation has been linked to anxiety, distress, depression, isolation and quality of life levels that match those of patients receiving treatment [12, 17–19, 31, 32]. Indeed, psychological adjustment has been described as more difficult in these cancers than physical effects [12]. Unfortunately, the very nature of chronic haematological malignancies means patients attend clinic infrequently, or less often those with acute subtypes, so may have little face-to-face time with clinicians [19], reflecting fewer opportunities for HCPs to identify difficulties, provide reassurance and facilitate interventions.

Interestingly, patients with cancer have been described as experiencing ‘a journey of never-ending making sense’ as they attempt to regain control over their lives, despite changes in their disease and treatment [33]; a compelling perspective in the context of chronic blood cancers. A further interesting notion pertinent to the inherent uncertainty associated with blood cancers is that discussions about the future and prognosis should adopt an individualized, sensitive and honest approach that achieves a balance between hope and a realism [34–37]. Not a new idea, this concept gained importance in the last decade and may protect patients from over-optimism regarding prognosis, as was noted in our study and is recognised to exist more generally among HCPs providing cancer care [38, 39]. It may also improve preparedness for disease progression or relapse, and the need for (more) treatment, or indeed end of life care, where this situation arises.

As noted, patients with chronic blood cancers do not always commence treatment immediately at diagnosis, but may instead be observed, only receiving treatment at disease progression or when they become (more) physically symptomatic; a concept many patients found difficult to process and a factor that contributed to their anxiety. Such anxiety is perhaps unsurprising, however, as preventing delayed cancer diagnosis is at the forefront of NHS policy and, combined with early treatment, is recognised as a means of maximising survival [40, 41]. In this context, and as similar initial management strategies may also occur in other cancers (e.g. active surveillance in prostate cancer [42]), raising awareness among the public that such pathways are evidence-based and set within national guidance may be helpful.

In line with our findings, other studies note a pre-diagnostic lack of knowledge about haematological malignancies, and an ongoing lack of understanding about these diseases, although there was recognition that they differed from other cancers [5, 12, 43]. The patients we interviewed who had been diagnosed with myeloma described what appeared to be particularly difficulty pathways compared to those with the other subtypes of interest, perhaps because disease progression was almost inevitable in myeloma [18]; complications (e.g. fractures and renal failure) often occurred before and after diagnosis [44]; and quality of life and physical functioning was considered more problematic than for other blood cancers [45]. This group of patients is also reported to have limited understanding of their diagnosis, compared to levels of comprehension among people with other cancers [15].

Worryingly, patients in a UK study of CLL noted how their doctors do not always seem to fully appreciate how they feel, or are affected by their cancer [17]. The research we present here, however, in conjunction with that of others, has clearly shown that problems (e.g. psycho-social and information needs) may be significant and enduring [12, 17–19, 31, 32]. This is important in the context of clinical practice and health policy (including survivorship), in order to ensure the unmet needs and challenges experienced by patients with chronic blood cancers are not overlooked by HCPs, simply because their disease is less acute than other haematological malignancies, and more long-term. It is also important that further research takes place examining the extent to which HCPs are aware of anxieties and distress; and the availability and effectiveness of interventions to address this.

To our knowledge this is the first study focusing solely on the experiences of patients with chronic haematological malignancies. Purposive sampling ensured information rich participants were included [22], thereby aligning with the concept of information power [24, 25]. The analytical process involved two experienced researchers and quality was ensured via ongoing engagement with the data and reflexive interpretation [25]. Findings are comprehensively described and provide new insights into an important, under-researched area, which highlights significant challenges for patients. Our results are likely to be transferable [46] within the UK and countries with similar health-care services, and to other chronic cancers/conditions. Despite significant efforts, we were unsuccessful in recruiting interviewees ethnic minority backgrounds, and future dedicated research is required in this area. Finally, we interviewed patients who consented to further contact via the HMRN Partnership, thus did not capture the experiences of those too ill to be approached.

## 5. Conclusion

Many participants lacked knowledge about chronic haematological malignancies. HCPs acted to reassure patients about their diagnosis, and while this was appropriate and effective for some, it was less so for others, as the cancer-impact involved struggling to cope with ongoing, uncertainty, anxiety, distress and a shortened life-span, which could be more burdensome than any physical symptoms.

## Supporting information

S1 File (DOCX)
